# Biological Effects of Listeriolysin O: Implications for Vaccination

**DOI:** 10.1155/2015/360741

**Published:** 2015-03-22

**Authors:** K. G. Hernández-Flores, H. Vivanco-Cid

**Affiliations:** ^1^Instituto de Investigaciones Medico-Biológicas, Universidad Veracruzana, 91700 Veracruz, VER, Mexico; ^2^Doctorado en Ciencias Biomédicas, Centro de Investigaciones Biomédicas, Universidad Veracruzana, 91000 Xalapa,VER, Mexico; ^3^Facultad de Medicina “Dr. Porfirio Sosa Zarate”, Universidad del Valle de México, Campus Villa Rica, 94299 Boca del Río, VER, Mexico

## Abstract

Listeriolysin O (LLO) is a thiol-activated cholesterol-dependent pore-forming toxin and the major virulence factor of *Listeria monocytogenes* (LM). Extensive research in recent years has revealed that LLO exerts a wide array of biological activities, during the infection by LM or by itself as recombinant antigen. The spectrum of biological activities induced by LLO includes cytotoxicity, apoptosis induction, endoplasmic reticulum stress response, modulation of gene expression, intracellular calcium oscillations, and proinflammatory activity. In addition, LLO is a highly immunogenic toxin and the major target for innate and adaptive immune responses in different animal models and humans. Recently, the crystal structure of LLO has been published in detail. Here, we review the structure-function relationship for this fascinating microbial molecule, highlighting the potential uses of LLO in the fields of biomedicine and biotechnology, particularly in vaccination.

## 1. Introduction

Listeriolysin O (LLO) belongs to the family of cholesterol-dependent cytolysins, which contains more than 20 pore-forming toxins produced by different bacterial species [1]. Recently, the crystal structure of LLO has been published in detail [2]. The whole molecule is a rod-like protein with four distinct domains, referred to as D1 to D4 [2]. Structurally, D1 contains a five-stranded *β*-sheet and is surrounded by six *α*-helices [2]. D1 also contains a key sequence for the LLO function, a signal sequence of 25 amino acids [3]. The signal sequence is cleaved off during the protein secretion and therefore is not present in the mature molecule [2]. Another important region, comprising amino acids 39–51 of D1, is known as a PEST-like sequence (P, Pro; E, Glu; S, Ser; and T, Thr) [2]. The PEST sequence is not necessary for the hemolytic activity but it is critical for the phagosomal escape and to establish the* Listeria monocytogenes* (LM) infection* in vivo* [4, 5], so it is essential for virulence and intracellular compartmentalization. Due to the presence of six prolines, the PEST sequence presents an arrangement known as polyproline type II (PPII) [2]. PPII has been involved in a regulatory role in the host cytosol, inhibiting or preventing LLO oligomerization and pore formation in this cellular compartment [2].

D2 is a key sequence to connect D1 to D4. D2 consists in four *β*-strands and is connected to D4 through a glycine linker [2, 6], at residue 417 [2]. In the murine model of LM infection, the anti-LLO immune response is directed to D2, which has been shown to be a highly immunogenic region and a key target of the host immunity [7]. The immunodominant epitope (91–99 aa) in the D2 sequence is recognized by CD8+ T cells in the H-2^d^ genetic background (BALB/c mice strain) [7].

**Figure 1:**
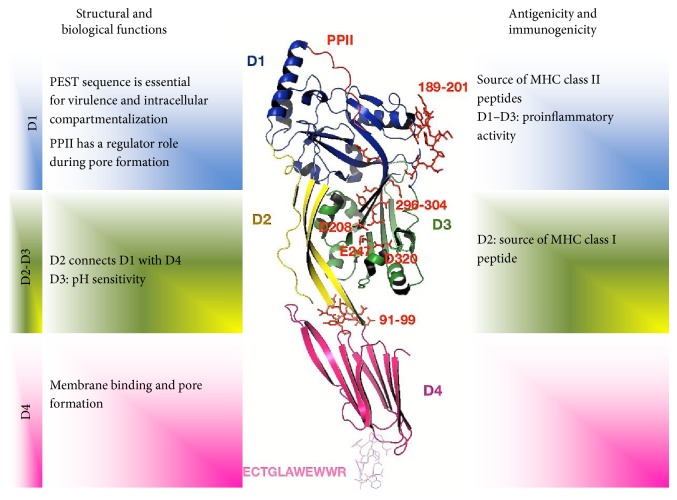
A structure-function relationship model of Listeriolysin O (LLO). LLO domains are represented in a different color: domain 1 blue, domain 2 yellow, domain 3 green, and domain 4 pink. Key residues or sequences for biological activity or immunogenicity are highlighted in red color. The LLO structure-function model was generated based on the crystal structure reported by Köster et al. [2] with PYMOL program.

D3 is formed by a five-stranded antiparallel *β*-sheet, which is surrounded by six *α*-helices [2]. Previously, it has been described that three residues in the D3 region are important as a pH-sensor, comprising D208, E247, and D320 residues [8]. The recently published crystal structure shows that one Na+ and a water molecule are necessary to mediate the interactions of D208, E247, and Y206 from the central *β*-sheet with D320 and K316 from the second membrane-inserting helix bundle [2]. LLO conformation is regulated by temperature and pH-dependent mechanisms [8]. The pH sensor triggers the denaturation of LLO at neutral pH [8]. D4 is the most wide studied region in the LLO structure. D4 has eight *β*-sheets, which are organized forming a *β*-sandwich structure [2]. The major feature of the D4 is the presence of a highly conserved structural motif of 11 residues (ECTGLAWEWWR) in the C terminal region that is considered crucial for membrane binding and cytotoxic activity [1] (Figure 1).

## 2. LLO and Cell Death Induction: From Cytotoxicity to Apoptosis

It has been described that LLO is highly lytic for the nucleated cells and it can induce a wide range of different cell death types [9–23]. The most studied mechanism is the cytolysis in different eukaryotic cells, including red blood cells, primary immune cells, and a wide spectrum of cell lines. By hemolysis assays and electron microscopy, it has been observed that LLO is able to induce lysis in red blood cells from human, different animal species at concentrations as low as 5 ng/mL [9], and primary immune cells such as bone marrow derived macrophages (BMM) [10]. The lytic activity is also exerted on other cells, such as A20, a B cell lymphoma [9], Caco-2 cells [11], J774 [12], Jurkat [13], and HepG2 cells [14]. The lytic doses are different between cell types, ranging from 0.5 to 200 nM of LLO. Cytotoxicity occurs rapidly during the first minutes after incubation with LLO. Whereas the cytotoxic activity of LLO is evident in short times and at high doses, the induction of apoptosis occurs at later time and with sublytic concentrations. During the infection by LM, the bacteria induce cellular apoptosis in the spleen, lymph nodes, liver, and brain [15–17].

As a purified or recombinant protein, LLO induces cellular apoptosis in some primary immune cells, such as bone marrow dendritic cells (BMDC), BMM [18], primary T cells [19], cell lines such as CB1, a murine dendritic cell line [20], and A5, a T cell hybridoma [21]. Activated but not resting T cells are susceptible to the proapoptotic effect of LLO [19]. The mechanism of apoptosis induction mediated by LLO on activated T cells includes two events: one mediated by activation of caspase-3 and caspase-6 [19]. Caspase activation depends on the granzymes expression [22]. A second mechanism which is LLO-dependent but caspase-independent induces the exposure of phosphatidylserine and the loss of the mitochondrial membrane potential [19]. The mechanisms of apoptosis induction for other cell populations different from T cells have not been described. The* in vitro* results of apoptosis induction mediated by LLO are in agreement with* in vivo *observations. Injection of purified LLO into the footpads of mice led to TUNEL positive cells in the peripheral cortex and paracortex of the draining popliteal lymph node but not the more distal inguinal lymph node, confirming the proapoptotic activity of LLO* in vivo* [19].

Recently, it has been described that LLO is able to induce other cellular activation pathways culminating in apoptosis induction. During LM infection in the P388D1 cell line, the bacterium induces the expansion of the endoplasmic reticulum (ER) and initiates a stress response to unfolded proteins (unfolded protein response or UPR). Induction of ER stress response is dependent on the production of LLO. LLO-deficient LM (LMΔhly) cells are not able to induce UPR. P388D1 cells stimulated with recombinant LLO reproduce UPR. Some activation markers of UPR increase after cell treatment with LLO or LM infection, such as the expression of protein disulfide isomerase, the processed form of activating transcription factor 6, phosphorylation of the *α*-subunit of eukaryotic translation initiation factor-2, increase of the spliced X-box binding protein-1, and high expression of immunoglobulin binding protein [23].

## 3. Innate Immunity to LLO

The immune system is the collection of tissues, cells, and molecules that protects the body from numerous pathogenic microbes and toxins in our environment. The defense against microbes and microbial molecules includes two general types of responses: innate immunity and adaptive immunity. The innate immune system consists of diverse mechanisms, tissues, cells, and proteins that are always present and ready to mobilize and fight against pathogens and microbial molecules at the site of infection. The main components of the innate immune system are physical epithelial barriers, granulocytes such as neutrophils, eosinophils, mast cells, monocytes, and antigen presenting cells such as macrophages and dendritic cells (DCs), natural killer cells (NK cells), and circulating plasma proteins [24]. The innate immune cells utilize germ-line coded receptors known as pattern recognition receptors (PRRs) which recognize highly conserved molecules and motifs in the microbial structures known as pathogen-associated molecular patterns (PAMPs) [25]. Upon PAMPs recognition, PRRs expressed by innate immune cells trigger proinflammatory and antimicrobial responses by activating different intracellular signaling pathways and transcription factors [26].

In addition to the detrimental cytolytic and proapoptotic activities, LLO has been described as PAMP, which is recognized by different innate immune cells, such as peritoneal macrophages [27], BMM [28], murine DCs [29], bone marrow derived mast cells (BMMCs) [30], basophilic leukemia cell line [30], NK cells [31], endothelial cells [32], human neutrophils [33], and human peripheral blood mononuclear cells (PBMCs) [34]. In all these cells, LLO induces a wide range of inflammatory mediators such as IL-1*α*, IL-1*β*, IFN-*γ*, TNF-*α*, IL-6, iNOS, IL-10, and IL-12.

It has been shown recently that wild type LM, but not LM lacking LLO or expressing a non-pore-forming LLO, stimulates a strong IL-1*β* production in human PBMCs. Also the stimulation of human PBMCs with purified LLO induces the IL-1*β* secretion. Both infection with LM and stimulation with LLO induce the activation of a NOD-like receptor member: NLRP3, the best characterized inflammasome family member, which is critical for IL-1*β* production by PBMCs [34]. Interestingly, LLO induce K+ efflux in HeLa and THP1 human cell lines through pore formation at the cell membrane. The K+ efflux initiates cascade signals leading two different events: desphosphorylation of histone H3 and inflammasome activation with caspase-1 and IL-1*β* production [35]. Also, the membrane damage on the surface of BMM by recombinant LLO stimulates the caspase-7 cleavage with a subsequent cytoprotective response [36].

Different mechanisms are involved in the inflammatory response induced by LLO. The role of LLO as a toll-like receptor 4 (TLR-4) agonist is controversial. BMM from C3H/HeJ mice (LPS-hyporesponsive mice, which present a point mutation in the TLR-4 gene), do not respond with proinflammatory cytokine gene upregulation after stimulus with LLO [28]. In contrast, other research groups have reported evidence of TLR-4 independent activation pathways [29].

Other activation mechanisms described for LLO include intracellular calcium oscillations. A cytosolic Ca^2+^ elevation in BMMCs in response to LLO has been described. Pretreatment of LLO with cholesterol inhibits the Ca2 influx, suggesting that this phenomenon is pore-forming dependent. Calcium influx induces degranulation, activation, and release of TNF-*α*. The TNF-*α* production involves the translocation of the nuclear factor of activated T-cells (NFAT), a key transcription factor for gene expression [30].

Another mechanism that may explain the cellular activation induced by LLO in murine macrophages is the aggregation of lipid rafts. Rafts aggregation is dependent of oligomerization of LLO. This cellular event induces tyrosine phosphorylation events and the accumulation of surface molecules such as CD14, a protein anchored to the membrane by a glycosylphosphatidylinositol tail and coreceptor along with TLR4 for the detection of bacterial lipopolysaccharide (LPS) [37].

## 4. Adaptive Immunity to LLO

Adaptive immunity is mediated by antigen-specific immune mechanisms. The adaptive response and its specificity may be acquired following to the exposure to antigens, after the onset of infectious disease, by asymptomatic carriage of the pathogen, by harboring an organism with a similar structure (cross-reacting) or by vaccination. The white blood cells responsible for adaptive immunity include two different lymphocytes: T cells and B cells. Typically, B cells mediate humoral responses through the antibodies production. The main biological functions of antibodies are neutralization, complement activation, cellular cytotoxicity antibody dependent, and opsonization. Antibodies protect the host mainly against extracellular antigens. In contrast, T cells mediate cellular immune responses and recognize foreign antigen only when presented by major histocompatibility complex (MHC) molecules on the cell surface of antigen presenting cells (APCs) [38, 39].

In addition to the innate immune mechanism activated by LLO, the toxin is a prominent and primary target protein of the host's acquired immune system [40, 41]. In the mouse model of infection, it has been shown a potent cellular response against LLO. The adaptive cellular response to LLO differs in different murine genetic backgrounds such as H-2b, H2-d, and H2-k. All these mouse strains differ in the haplotypes of MHC molecules, which in mouse are codified in the* H-2* loci.* H-2* is a complex of genetic loci on chromosome 17 of the mouse. Allelic differences in the* H-2* complex affect host resistance to infection and cellular immune responses against specific antigens [42].

For example, during the primary infection of BALB/c mice by LM, the cellular response is detectable between day 2 and day 4 after infection and reaches a peak production on day 6 [43]. The adaptive immune response is mainly mediated by CD8+ T cells, which is key in the control of the infection and the long-lasting immune memory response [7]. In adoptive transfer experiments, it has been shown that CD8+ T cells specific for LLO are protective* in vivo* against LM infection [44].

CD8+ T cell response is mainly focused on the immunodominant epitope LLO 91−99 produced in infected H-2d mice [7]. In contrast, a weak CD4+ T cell response to LLO is observed in this genetic background. The CD4+ T cells response is mainly focused on LLO 189–200 epitope [45].

The infection of C57BL/6 mice with LM induces a strong CD4+ T cell response with an elevated frequency of LLO-specific cells but a poor anti-LLO CD8+ T cell response.

In this particular genetic background, the immunodominant peptide for CD4+T cells includes the LLO 190–201 amino acids. Interestingly, the CD4+ T cell response against this antigenic region is also shared and conserved in BALB/c mice. Moreover, the CD8+ T cell response is directed to the 296–304 amino acid sequence [45]. Similarly, the infection of C3HeB/FeJ mice (H-2k genetic background) with LM induces a dominant CD4+ T cell response directed to the LLO 215–234 peptide [46].

**Figure 2:**
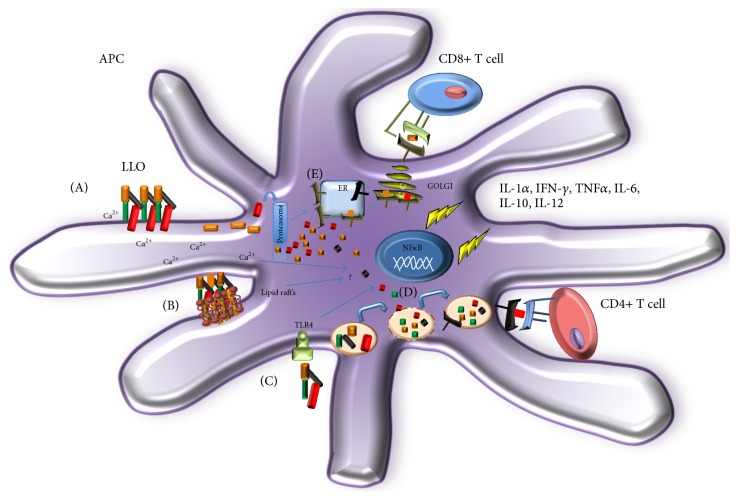
Innate and acquired immune response to LLO. (A) Pore forming activity of LLO induces cytosolic Ca^2+^ elevation in immune cells, which results in degranulation, activation, and release of proinflammatory molecules. (B) The binding of LLO on cell membranes induces lipid rafts aggregation, which is involved in cellular activation. (C) LLO has been described as a pathogen associated molecular pattern (PAMP), which is recognized by toll like receptor 4 (TLR-4), which results in NF-*κ*B activation and gene modulation. As a recombinant antigen LLO traffics very efficiently to endosomal (D) or cytosolic compartments (E) promoting a highly efficient presentation to CD4+ and CD8+ T cells.

As a recombinant antigen, LLO is also a highly immunogenic molecule. A very robust* in vivo* cellular response is observed after immunization with wild type LLO into the footpads of C57BL/6 mice [18].* In vitro*, the processing and presentation of LLO by murine APCs occur very rapidly. During the first 15–30 minutes, LLO is bound, internalized, and presented by APCs to induce the proliferation of LLO specific CD4+ and CD8+ T cell hybridomas. The presentation and activation of CD4+ T cell response is highly efficient, even at very low concentrations of LLO, such as the picomolar or femtomolar range [18]. A summary of the main activation pathways and the adaptive immunity described for the LLO molecule is shown in Figure 2.

## 5. Potential Biomedical Uses for LLO as an Adjuvant and Carrier Molecule

In experimental animal models, LLO has been evaluated as an adjuvant in vaccination to induce protection against pathogens, allergies, and tumors. Administration of killed LM together with purified LLO induces protective immunity against the bacteria in mice [47]. LLO also has been used in combination with p60 antigen and inactivated LM (iLM).

p60 is a 60-kDa extracellular protein produced by LM and acts as a murein hydrolase required in the last step of cell division [48]. Recombinant p60 induces proinflammatory cytokines and modulates host immune responses [49]. The vaccine showed the highest titers of anti-LM antibodies compared to the immunized mice with iLM alone, p60+ iLM, or LLO+ iLM. The presence of LLO in the experimental vaccine induced high levels of IFN-*γ*, confirming LLO as the major contributor to induce this cytokine in immunized mice [50]. In an ovalbumin- (OVA-) induced allergic rhinitis mouse model, LLO facilitates a polarization toward a Th1 profile, by inducing the production of inflammatory cytokines such as IFN-*γ*. The intranasal challenge with recombinant LLO, in combination with OVA, suppressed the allergic responses, reduced the anti-OVA IgE titers in serum and the eosinophil infiltration in the nasal mucosa [51]. Using a DNA vaccine strategy bearing a tumor antigen, the E7 antigen from the human papilloma virus (HPV) either alone or in combination with LLO, the effective adjuvant property of LLO to enhance the antitumoral protection has been demonstrated. In this model, LLO acts as an adjuvant to enhance both CD4+ and CD8+ T cell responses. The genetic fusion of LLO to E7 antigen led to inducing the enhancement of E7-specific CD8+ T cell responses, suggesting a dual role for LLO like an adjuvant and also as an effective carrier molecule for antigen delivery to the MHC class I pathway. The fusion of LLO to E7 was not required to augment E7-specific CD4+ T cell responses [52].

LLO has also been used in the treatment of other experimental tumor models, such as follicular lymphoma and head and neck cancers. A chemically conjugated LLO with the 38C13 lymphoma Id protein (38Id-LLO) was evaluated as a vaccine and compared with the same antigen conjugated to keyhole limpet hemocyanin (KLH). 38Id-LLO induced very potent humoral and cellular responses and was very effective in inducing antilymphoma protection in immunized mice. Mice previously vaccinated with 38Id-LLO survived after a lymphoma challenge. As in the previously described models, conjugation to LLO polarized the T cell responses toward a Th1 profile, promoting a high titer of IgG2a anti-idiotype antibodies [53]. In another murine cancer model, a DNA vaccine, which consists of the whole LLO sequence fused to the fetal liver kinase 1 (Flk1), a murine homologue of vascular endothelial growth factor receptor 2, was very effective in inducing tumor regression and a robust antitumoral immune response, compared with a DNA vaccine that contains Flk1 alone [54].

Other scientific evidence of LLO as a carrier/adjuvant molecule comes from recent studies with different model antigens. LLO can enhance the internalization, processing, and presentation of immunodominant peptides from hen-egg white lysozyme (HEL). A chimeric fusion protein that contains the 45–65 peptide of HEL protein fused to the aminoterminus region of LLO enhances by approximately 1000-fold the efficiency of presentation of the peptide to HEL-specific CD4+ T cell hybridomas, compared to the peptide alone [18]. Another experimental strategy consists of the use of sensitive pH liposomes that contain LLO. In this approach LLO is used as a vaccine adjuvant, taking advantage of its pore forming property on the cell membrane to provide cytosolic access for antigens in APCs. Liposomes that contain LLO have been used to deliver OVA [55] and lymphocytic choriomeningitis virus (LCMV) nucleoprotein (NP) [56] into the cytosolic pathway and to promote the degradation of these antigens in the cytosolic space. Furthermore, the observed antigen presentation for OVA and NP occurred by the conventional MHC class I pathway. LLO-liposome-mediated OVA immunization in mice induced robust OVA-specific cytotoxic T lymphocyte (CTL) activity [57]. Immunization of mice with LLO-liposomes containing NP generated a high frequency of NP-specific CD8+ T cells and protected against a lethal intracerebral challenge with a virulent strain of LCMV [56]. Thus, the combination of liposomes and LLO facilitates the delivery of any macromolecules into the cytoplasmic space and promotes a highly efficient cytotoxic response.

## 6. Current State of LLO Mutants in Experimental Models: Future Adjuvants/Carrier Candidates for Human Vaccination?

Given that LLO is a very potent toxin with adverse effects in both* in vitro* and* in vivo* biological systems, different technical approaches have been described for truncating or mutagenizing this molecule and studying its biological functions in the absence of its cytotoxic activity. Some examples of LLO mutants generated by different research groups and the resulting effects on its biological activities are shown in Table 1.

Based on this evidence, it is clear that the LLO property of being a key target of the host innate immune response (PAMP molecule) is independent of its cytolytic activity.

Kohda et al. previously described that truncated forms of LLO, including domains 1–3 but not domain 4, are capable of inducing* in vitro* the IFN-*γ* production by mouse spleen cells. These results confirm that the key region for the innate immune recognition and the induction of proinflammatory cytokines is located in domains 1–3, in contrast to the role of domain 4 in binding to cholesterol membranes. Moreover, pretreatment of LLO with free cholesterol does not affect inflammatory activity but reduces the cytotoxicity, suggesting that the pore-forming property of cellular membranes is dispensable for the inflammatory induction mediated by LLO [12].

A detoxified, nonhemolytic form of LLO (dtLLO) has been described recently [29], as an effective adjuvant in tumor immunotherapy dtLLO was generated by site-directed mutagenesis, introducing mutations in three points, C484A, W491A, and W492A, in the cholesterol binding domain, reducing its lytic activity by 99.9%. Wallecha et al. demonstrated that dtLLO induces the upregulation of proinflammatory cytokine mRNAs and the overexpression of costimulatory molecules in dtLLO-stimulated BMDCs from both wild type (C57BL6) and TLR-4^−/−^ deficient mice [29]. Immunization of mice with genetically fused E7 antigen from HPV to dtLLO or a combination of dtLLO mixed with the E7 antigen enhances the immune response to E7 protein and induces a reduction of the tumor burdens, and more than 50% of the mice were free of tumors at the end of the experiments [29].

Carrero et al. have shown that mutation of the tryptophans to alanines at both residues 491 and 492 (LLO W491-492A) or at only residue 492 (LLOW492A) led to a reduction in hemolytic activity of ~95–99.5% and in cytolytic activity to nucleated cells. Interestingly, the significant reduction in terms of cytotoxicity is not accompanied by a significant reduction in the uptake (~15–20%), binding, catabolism, processing, and presentation of immunodominant peptides to CD4+ or CD8+ T cells from these LLO mutants, confirming that antigenicity of LLO is also independent of its cytotoxic property [18].

## 7. Conclusions

Vaccination is the most efficacious and valuable tool in the prevention of infectious diseases, and also it could be an important strategy for immunomodulation of some chronic pathologies such as allergies, autoimmunity, and cancer. Vaccines require optimal adjuvants and carrier molecules to achieve effective long term protection. With the knowledge gained in recent years about the structure, biological activities, and immune response to LLO, it has opened a potential and attractive use of this molecule in the applied research field. Of all the biological activities demonstrated* in vitro* or* in vivo* by LLO, there are two key properties: first its proinflammatory capacity and second its potential for delivering antigens toward specific intracellular compartments in APCs.

The ability of LLO fusion proteins to deliver antigens toward endosomal and cytosolic compartments for presentation by both MHC class I and class II molecules is an attractive approach to use LLO as an effective carrier molecule in vaccination. As a recombinant antigen LLO releases endosomal contents into the cytosol, providing a possible mechanism for entry of endocytosed antigens into the MHC class I pathway [18].

In looking for safer molecules without the detrimental effects of LLO wild type, different technical approaches have generated multiple LLO-derived toxoids. Some important lessons we have learned from the manipulation of LLO structure are close to being applied in the vaccination field. First, now we know that for some LLO mutants the proinflammatory capacity is independent of its cytolytic activity [12, 29]. This is a remarkable advantage for a potential safe use of noncytolytic LLO mutants in human vaccination. However, the current information about LLO mutants as adjuvants or carrier molecules is limited to few examples, and extensive research is required to characterize the other LLO toxoids which have been generated and described in the literature. Finally, noncytolytic LLO mutants still maintain the binding capacity to the cell membranes with high affinity; they are catabolized, processed, and presented efficiently by APCs to CD4+ or CD8+ T cells. Proteins or antigenic peptides fused to these noncytolytic LLO mutants represent a powerful strategy to use these molecules as an ideal vaccine adjuvant/carrier to boost both humoral and cellular responses.

## Figures and Tables

**Table 1:** Summary of single point mutations and truncated LLO forms and the relationship on biological activities.

	LLO mutants	Effects of mutations on cell death	Effects of mutations on proinflammatory activity	Effects of mutations on LLO Immunogenicity	Reference
Single amino acids substitutions	LLO C484A	A decrease of 28% in the hemolytic activity	ND	ND	[58]
	LLO C484S	A decrease of 85% in the hemolytic activity	ND	NA	[20, 58]
	LLO W491A	A decrease of 95% in the hemolytic activity and abolished capacity to induce apoptosis	ND	ND	[20, 58, 59]
	LLO 415 (domains 1–3, no nomain 4)	Loss of cytolytic and lethal activities	ND	ND	[59]
	LLO W492A	A decrease of 99% in the hemolytic activity	ND	Retains the ability to be efficiently presented by APC's	[18, 58, 59]
	LLO W491-492A	A decrease of 99.5% in the hemolytic activity	ND	Retains the ability to be efficiently presented by APC's	[18]
	LLO W489A	Cytolytic activity diminished	ND	ND	[59]
	dtLLO (3 punctual mutations: C484A, W491A, and W492A.	No hemolytic activity	Retain the proinflammatory activity	ND	[29]
	LLOA40W, LLOS44D, LLOS44E, LLO D394W	Increased the hemolytic activity	ND	ND	[2]
	LLOK175E, LLOE262K, LLOS176W, LLOD4 (domain 4)	No hemolytic activity	ND	ND	[2]
	LLOE262W	No effect on cytolytic activity	ND	ND	[2]
	LLON230W	A decrease of 50% in the hemolytic activity	ND	ND	[2]

LLO truncated forms	Deletion of PPII region: LLODPPII.	Increased the hemolytic activity	ND	ND	[2]
	rLLO493, rLLO482, rLLO415; (Forms deficient of C-terminal region)	No hemolytic activity	Retain the proinflammatory activity	ND	[12]
	rLLO416–529 (domain 4 alone)	No hemolytic activity	No influence on proinflammatory activity	ND	[12]
	rLLO59–415 include the sequence 59–415aa of LLO)	No hemolytic activity	Retain the proinflammatory activity	ND	[12]

ND = not determined.
